# First Peek into the Transcriptomic Response in Heat-Stressed Tomato Inoculated with *Septoglomus constrictum*

**DOI:** 10.3390/plants13162266

**Published:** 2024-08-15

**Authors:** Viktor Szentpéteri, Eszter Virág, Zoltán Mayer, Nguyen Hong Duc, Géza Hegedűs, Katalin Posta

**Affiliations:** 1 Department of Microbiology and Applied Biotechnology, Institute of Genetics and Biotechnology, Hungarian University of Agriculture and Life Sciences, 2100 Gödöllő, Hungary; szentpeteri.viktor@uni-mate.hu (V.S.); mayer.zoltan@uni-mate.hu (Z.M.); hongduc.real@gmail.com (N.H.D.); 2 Agribiotechnology and Precision Breeding for Food Security National Laboratory, Institute of Genetics and Biotechnology, Hungarian University of Agriculture and Life Sciences, 2100 Gödöllő, Hungary; 3 Institute of One Health, University of Debrecen, 4032 Debrecen, Hungary; eszterandreavirag@gmail.com; 4 Research Institute for Medicinal Plants and Herbs Ltd., 2011 Budakalász, Hungary; 5 Department of Information Technology and Its Applications, Faculty of Information Technology, University of Pannonia, 8900 Zalaegerszeg, Hungary; hegedus.geza@zek.uni-pannon.hu

**Keywords:** AM fungi, tomato, heat stress, RNA sequencing, transcriptomic analysis

## Abstract

In this study, we report the interaction between an arbuscular mycorrhizal fungus, *Septoglomus constrictum*, and tomato plants under heat stress. For the first time, this interaction was studied by Illumina RNA-seq, followed by a comprehensive bioinformatic analysis that investigated root and leaf tissue samples. The genome-wide transcriptional profiling displayed fewer transcriptomic changes in the root under heat-stress conditions caused by *S. constrictum*. The top 50 DEGs suggested significant changes in the expression of genes encoding heat-shock proteins, transporter proteins, and genes of phytohormone metabolism involving jasmonic acid signalling. *S. constrictum* induced the upregulation of genes associated with pathways such as ‘drought-responsive’ and the ‘development of root hair’ in the root, as well as ‘glycolipid desaturation’, ‘intracellular auxin transport’, and ‘ethylene biosynthesis’ in the leaf. The pathways ‘biotin biosynthesis’ and ‘threonine degradation’ were found in both investigated tissue types. Expression analysis of transcription factors showed 2 and 11 upregulated transcription factors in heat-stressed root and leaf tissues, respectively. However, we did not find shared transcription factors. Heat-stressed arbuscular mycorrhizal plants suffered less oxidative stress when exposed to high temperatures. Colorimetric tests demonstrated less accumulation of H_2_O_2_ and MDA in heat-stressed mycorrhizal plants. This phenomenon was accompanied by the higher expression of six stress genes that encode peroxidases, glutathione S-transferase and ubiquitin carboxyl-terminal hydrolase in roots and leaves. Our findings provide a new perspective on elucidating the functional metabolic processes of tomato plants under mycorrhizal-heat stressed conditions.

## 1. Introduction

Tomato (*Solanum lycopersicum* L.) is one of the most widely consumed fruits and is a key source of vitamins and bioactive compounds [1]. In addition to its economic and nutritional value, the importance of tomatoes is also highlighted by its well-researched genome, making it a perfect model organism for transcriptome analysis [2]. The cultivated tomato is diploid, contains 12 chromosomes, and has a ca 900 megabase (Mb) genome size; moreover, computational annotation supported by RNA sequencing data predicts the existence of 34,727 protein-coding genes [3]. Like many other terrestrial plants, tomato can form arbuscular mycorrhizal (AM) symbioses, which involve the formation of a unique structure between the roots of most land plants and AM fungi [4]. Establishing and maintaining this symbiosis requires complex molecular interactions between the partner organisms and results in comprehensive changes in gene expression profiles [5,6,7]. Through these changes, AM fungi can increase the growth and vigour of host plants, even under adverse environmental conditions. Mycorrhizal plants are more likely to withstand temperature, drought, salinity and heavy metal toxicity than their nonmycorrhizal counterparts [8]. While the alleviating effects of AM fungi under cold stress have frequently been examined [9,10,11], their effect at high temperatures has rarely been observed [12,13,14]. With the increase in the global mean annual temperature [15] and the increasing frequency of heat waves, improving our knowledge in this field is becoming increasingly important to avoid economic losses in agriculture and guarantee food security [16]. Yield size and crop quality are especially endangered in the case of tomatoes due to their extreme sensitivity to heat stress, as it can lead to the abortion of flowers [17]. Besides harming the reproductive phase, heat stress affects vegetative growth. Biomass, internode length and photosynthesis rate are lowered as plants close their stomata to retain water. Thus, CO_2_ uptake is hindered [18,19].

Previous studies have shown that at the cellular level, heat stress induces several changes, including disruption of homeostasis, misfolding of proteins, failure of microtubule organization, damage to membranes, and accumulation of reactive oxygen species (ROS) at harmful levels [20,21,22,23]. Additionally, despite the positive roles of ROS in stress perception and signal transduction, their overproduction induces oxidative stress in cells, which damages lipids, proteins, and DNA [24]. Hydrogen peroxide (H_2_O_2_) is known as the primary reactive oxygen species in the redox regulation of biological activities. Supra-physiological concentrations of H_2_O_2_ lead to nonspecific protein oxidation as well as reversible and irreversible damage to biomolecules [25]. ROS attack or activation of lipoxygenases under stress conditions produce malondialdehyde (MDA). This may initiate defence signalling in case of a transient MDA accumulation, or trigger cell death when the accumulation is sustained. In order to control the level of aldehyde compounds, aldehyde dehydrogenase expression is induced to oxidize them to their corresponding carboxylic acids. These re-establish low cellular aldehyde levels to avoid cellular harm [26].

Improper protein folding creates stress in the endoplasmic reticulum, triggering the unfolded protein response [27]. Heat shock factors (HSFs), which play a central role in stress signal perception, regulate the expression of genes involved in the plant stress response and interact with heat shock protein (HSP) genes [28]. HSP overexpression in plants is positively correlated with thermotolerance [29]. 

In addition to the general heat stress response of plants, AM symbiosis also initiates cellular changes to maintain host survival at high temperatures [30]. Among the changes induced by AM fungi during heat stress, enhanced water use efficiency, retention capacity, and relative water content have to be mentioned. This is achieved by preserving the plasma membrane through the increased production of antioxidative enzymes and the generation of osmoprotectants like proline, trehalose, or glomalin [31]. Besides the cellular changes, the photosynthesis-related functions of mycorrhizal plants are also elevated [32]. The increased carbon phosphorous and nitrogen metabolism prime AM plants to better withstand and mitigate the adverse effects of heat stress [33]. AM-induced benefits have been shown to be closely connected to auxin, abscisic acid (ABA), gibberellin (GA), and brassinosteroid (BR) metabolism [34], these phytohormones play a crucial role in heat response and tolerance [35].

Transcriptome analysis is a powerful tool that provides insight into the molecular background of the stress response. Individually, mycorrhiza-induced transcriptomic changes [7,36,37,38] and the transcriptomic responses to heat stress [39,40,41,42,43] have been previously examined in tomato plants. However, the two factors together have not been discussed at the transcriptomic level. As the benefits to the growth, antioxidant system and hormonal metabolism of abiotic stress-exposed AM plants have been described previously, we hypothesized that mycorrhizal tomatoes would handle the heat shock-induced oxidative burst better by amplifying the general heat stress response or utilizing novel pathways, as the presence of *Septoglomus constrictum* primes the host on a transcriptomic level.

## 2. Results

### 2.1. Colonization Rate, Biomass and Water Potential Changes

AM colonization was well established in both mycorrhizal control (CAM) and mycorrhizal heat stress-treated (HAM) plants, with colonization rates of 41.35% and 67.87%, respectively, while it was undetectable in nonmycorrhizal control (C) and heat-stressed (H) plants. There was no significant difference in shoot dry weight between the C and H plants or between the CAM and HAM plants (Supplementary Table S1). Compared with the C plants, the HAM plants were the only ones that significantly differed in shoot dry weight, with a 27.5% higher value of 1.81 g. Regarding root dry weight, a significant difference (*p* < 0.05) was observed only in the CAM plants compared to the H plants, the weight difference was 17.9%. Heat stress induced a significant decrease in water potential in the leaves of both nonmycorrhizal and mycorrhizal (H and HAM) plants compared to that in the leaves of their untreated counterparts (C and CAM) with a decrease of more than 200% (CvsH) and 300% (CAMvsHAM), respectively.

### 2.2. Assessment of Stress Levels Induced by Hydrogen Peroxide and Malondialdehyde

**Figure 1:**
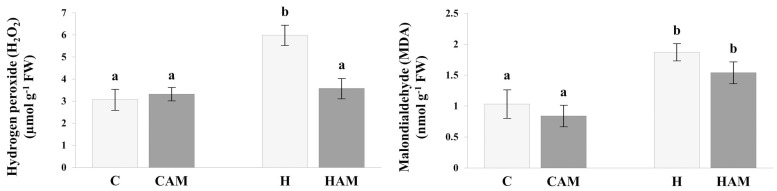
H_2_O_2_ and MDA concentration of leaves in control (C), mycorrhizal control (CAM), heat stress-treated (H), and mycorrhizal heat stress-treated (HAM) plants. Lowercase letters indicate statistical differences between treatments, according to one-way ANOVA combined with Tukey post hoc test (*p* < 0.05).

Compared with the control treatment, heat stress induced a significant accumulation of hydrogen peroxide (H_2_O_2_), the level of which increased twofold (*p* < 0.05) (Figure 1). However, this increase was not detected in the presence of *S. constrictum*, and the H_2_O_2_ concentration was close to the levels measured in the treatments without stress application. The malondialdehyde (MDA) concentration was also significantly increased by 81.5% under heat stress (CvsH). However, unlike in the case of H_2_O_2_, this increase (49.5%) was also present in mycorrhizal plants exposed to heat stress (CvsHAM) (Figure 1).

### 2.3. Genome-Wide Transcriptome Profiling and Differentially Expressed Genes

Transcriptomic changes between tissue types and treatments were demonstrated by the MDS plot (Supplementary Figure S1).

**Figure 2:**
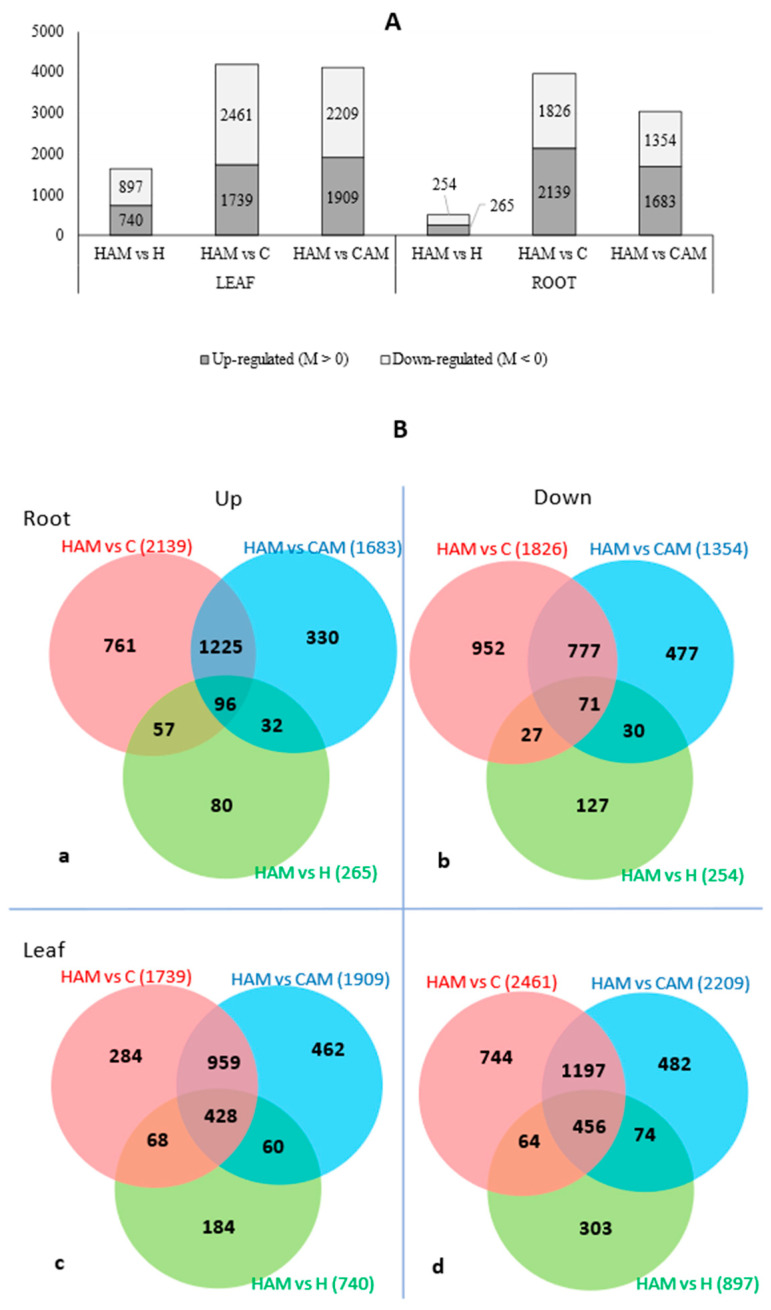
The number of differentially expressed (DE) features (probability > 0.9) of leaf and root paired samples (**A**). Venn diagrams (**B**) of up- and downregulated DEGs of root samples (**a**,**b**) and leaf samples (**c**,**d**). Independent subsets of genes of HAM vs. H sample pair as uniquely expressed genes were selected to be analysed in enrichment analysis (Fisher’s exact test), numerically: 80 (**a**), 127 (**b**), 184 (**c**), and 303 (**d**) genes were involved in the analysis.

Leaf and root samples of mycorrhizal heat stress-treated plants were compared to those of control, heat stress-treated, and mycorrhizal treatment plants to identify DEGs associated with the combined presence of these two factors. In the case of leaf samples, this resulted in 740 up- and 897 downregulated DEGs in the HAMvsH pairing, 1739 up- and 2461 downregulated DEGs in the HAMvsC pairing, and 1909 up- and 2209 downregulated DEGs in the HAMvsCAM pairing. In the case of the root samples, we found 265 up- and 254 downregulated DEGs in HAMvsH, 2139 up- and 1826 downregulated DEGs in HAMvsC, and 1683 up- and 1354 downregulated DEGs in HAMvsCAM (Figure 2A). In both tissue types, we identified a relatively low number of DEGs in HAMvsH compared to the other pairings. AM caused fewer transcriptomic changes in the roots under heat-stress conditions. There were no notable differences in the ratio of up- and downregulated genes in any sample or pairing. When focusing on the HAMvsH pairing to highlight unique AM-induced changes during heat stress, we found 80 upregulated and 127 downregulated genes in the roots, and 184 upregulated and 303 downregulated genes in the leaves.

**Figure 3:**
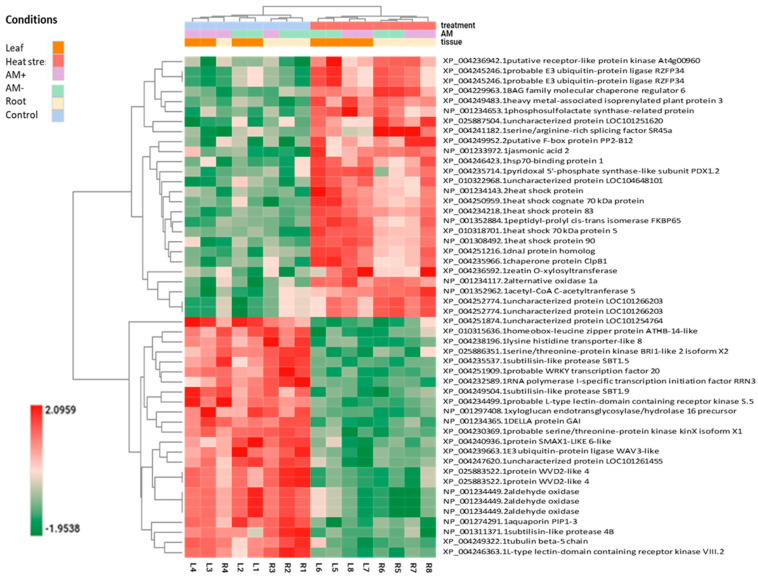
Heatmap of the top 50 differentially expressed genes (ranked by FDR) analysed and compared in all investigated leaf and root samples. The dendrograms added to the left and top sides are produced by a hierarchical clustering method that takes the Euclidean distance computed between genes (left) and samples (top) as input. Marks: treatments: red—heat stress; blue—control; purple—AM-inoculated; green—non-mycorrhizal; orange—leaf sample; cream—root sample; samples: L1, L2—control leaf samples; L3, L4—mycorrhizal leaf samples; L5, L6—heat stress-treated leaf samples; L7, L8—mycorrhizal and heat stress-treated leaf samples; R1, R2—control root samples; R3, R4—mycorrhizal root samples; R5, R6—heat stress-treated root samples; R7, R8—mycorrhizal and heat stress-treated root samples.

The top 50 DEGs of the combined (leaf and root) DEG datasets were visualized in a two-dimensional heatmap (Figure 3). Among the highly influenced genes were several genes encoding heat shock proteins or cognates, namely, heat shock protein (NP_001234143.2), heat shock cognate 70 kDa protein (XP_004250959.1), heat shock protein 83 (XP_004234218.1), heat shock 70 kDa protein 5 (XP_010318701.1), heat shock protein 90 (NP_001308492.1), and chaperone protein ClpB1 (XP_004235966.1). Hormone biosynthetic and hormonal response genes such as those encoding zeatin O-xylosyltransferase (XP_004236592.1), jasmonic acid 2 (NP_001233972.1), and DELLA protein GAI (NP_001234365.1) were among the top 50 DEGs, indicating a strong influence of AM on hormonal pathways under heat stress conditions. Additionally, two transporter genes, lysine histidine transporter-like 8 (XP_004238196.1) and aquaporin PIP1-3 (NP_001274291.1), were also found to be strongly influenced in our study.

### 2.4. Heat Stress Mitigation in Mycorrhizal Plants

After acquiring general information about DEGs in all treatments as a next step, we performed Gene Ontology (GO) term analysis of the DEGs identified uniquely in HAMvsH. From the point of view of heat stress, we considered the changes in this pairing to be the most relevant (Supplementary Figure S4). In the roots, 127 GO terms were downregulated and 80 were upregulated, while in the leaves, 303 terms were downregulated and 184 were upregulated. More terms showed a significant change in leaves than in roots. In the cellular component (CC) GO category, most DEGs were annotated to the nucleus, membrane, cytoplasm, organelle, and related subcategories in both tissue types. In roots, most genes were associated with the metabolic process, cellular process, catalytic activity, and binding in roots in the molecular function (MF) class. These terms contained DEGs from both the up- and downregulated groups. In leaves, the DNA-binding transcription factor activity, transcription regulator activity, ion binding, oxidoreductase activity, cation binding, and metal ion binding terms contained the most downregulated DEGs. In the biological process (BP) category, strong downregulation was observed in both roots and leaves, as 49 of the 82 terms were downregulated in either or both tissue types. Only upregulation was detected for the lipid metabolic process, response to external stimulus, cell communication, and cellular component organization terms. The terms upregulated in only the roots were lipid metabolism and response to external and biotic stimuli. Concerning the term generation of precursor metabolites and energy, we found opposing trends between the two tissue types, as this term was upregulated in the roots but downregulated in the leaves.

**Figure 4:**
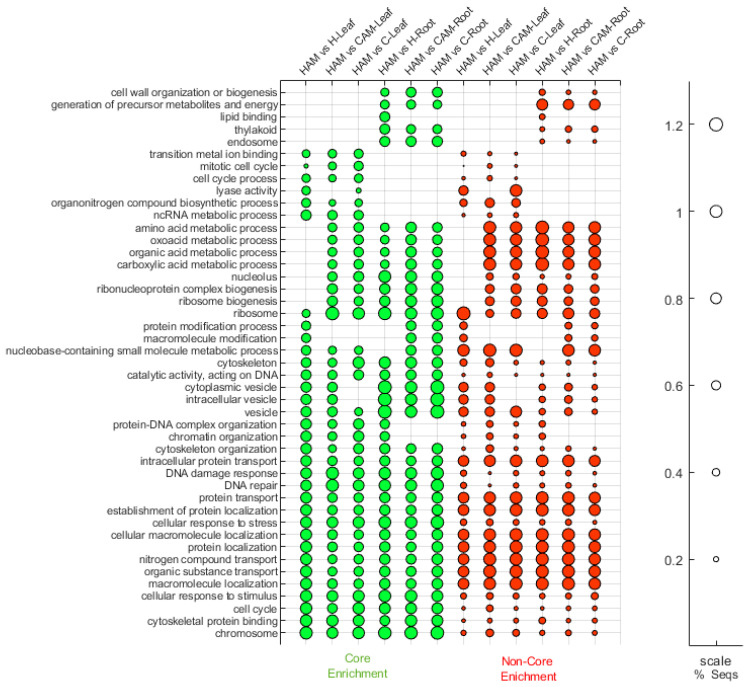
GSEA analysis of leaf and root samples. The cumulative top 30 GO categories of core and non-core enrichment by the NES of differentially expressed genes are shown in the figure.

Gene set enrichment analysis (GSEA) of the root and leaf samples was subsequently performed (Figure 4). In all three pairings, HAMvsC, HAMvsCAM, and HAMvsH, we found terms related to protein and nitrogen compound transport, localization, response to stress and stimuli, DNA damage and repair response, and cell cycle in all investigated leaf and root samples. We could isolate groups characteristic of only the root or leaf, those involved in leaf transition metal ion binding, the mitotic cell cycle, the cell cycle process, and lyase activity. Among the root GO terms, cell wall organization or biogenesis, generation of precursor metabolites and energy, lipid binding, thylakoid, and endosome were uniquely characteristic. We found groups that were lacking only in HAMvsH in leaves, namely amino acid, oxoacid, organic acid, and carboxylic acid metabolic processes, as well as nucleolus and ribosome-related GOs. However, the modification of proteins and macromolecules was highlighted only in leaves in the presence of AM (HAMvsH).

Based on these results, we investigated the GSEA data in depth in the HAMvsH category according to its highest specificity being observed using a 0.05 FDR filter (Supplementary Table S7). By analysing the top GO categories in the leaves, we selected genes related to cytoplasmic translation, translation regulator activity, response to hormones, and ubiquitin binding. In the roots, we selected genes related to photosynthesis and response to auxin to examine the core and non-core genes in detail (Supplementary Table S8). In the leaves, the less-enriched genes that we selected were related to photosynthesis, response to abiotic stimulus and antioxidant activity. Meanwhile, in the roots, we selected cytoplasmic translation and ubiquitin binding to examine the core and non-core genes in detail (Supplementary Table S8). Interestingly, the terms ubiquitin binding (GO:0043130) and cytoplasmic translation (GO:0002181) and several other ribosome-connected terms were among the top-ranked GO terms in the leaves but among the lowest-ranked GO terms in the roots. Several genes participating in auxin signalling were found in both tissue types, with genes involved in response to auxin and response to hormone terms at the forefront.

### 2.5. AM-Induced Changes That Occurred Only during Heat Stress

**Figure 5:**
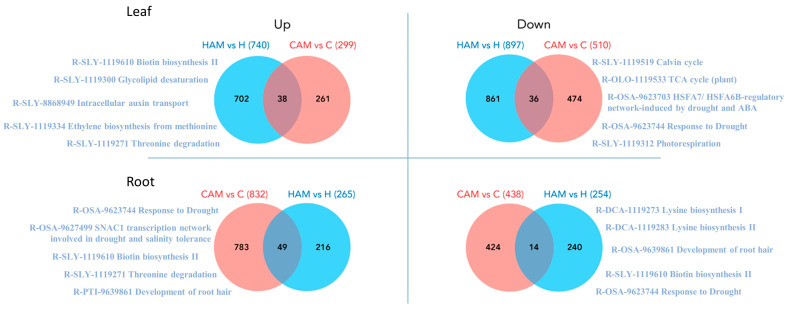
Venn diagrams of up- and downregulated DEGs of CAMvsC group compared to HAMvsH in root and leaf samples. Top five pathways are noted in each diagram according to the genes present in HAMvsH but absent in CAMvsC.

To identify the changes that represented the effects of mycorrhizal fungus during heat stress, we compared the CAMvsC group to HAMvsH and analysed only those DEGs that were unique to HAMvsH (Figure 5, Supplementary Table S6). In the roots, there were 216 upregulated and 424 downregulated genes, while in the leaves, there were 261 upregulated and 474 downregulated genes.

Pathway analysis of this list of DEGs was performed using the KEGG and Plant Reactome databases. The top five downregulated pathways in the roots revealed changes in lysine and biotin biosynthesis, root hair development, and drought response. Among the top five upregulated pathways in roots, we found two drought-responsive pathways, biotin biosynthesis, threonine degradation, and the development of root hair. In leaves, the downregulated pathways with the most genes were the Calvin cycle, the TCA cycle (plant), the HSFA7/HSFA6B regulatory network induced by drought and ABA, the response to drought, and photorespiration. The upregulated genes were associated with biotin biosynthesis II, glycolipid desaturation, intracellular auxin transport, ethylene biosynthesis from methionine, and threonine degradation (Figure 5).

We selected genes related to drought response, jasmonic acid signalling, and auxin signalling processes for a more in-depth analysis of the altered pathways. These terms were chosen either because they contained many DEGs that are important for the stress response or because they were highly induced in the L7,8 or R7,8 samples (Supplementary Figure S2; Supplementary Table S10).

**Figure 6:**
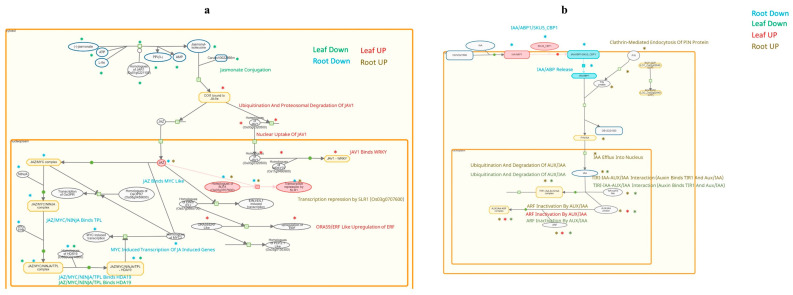
Jasmonate (**a**) and auxin (**b**) signalling pathways based on DEGs taking part in the processes found in HAMvH but not in CAMvsC comparison are marked with stars of different colour depending on the tissue type and direction of change (blue—downregulated gene in root; green—downregulated gene in leaf; brown—upregulated gene in root; red—upregulated gene in leaf).

Auxin signalling seems to be strongly influenced by the presence of mycorrhizae during heat stress (Figure 6). Genes involved in nuclear localization, such as those involved in the TIRI-IAA—AUX/IAA interaction, ubiquitination, and degradation of AUX/IAA, were upregulated in roots and downregulated in leaves. The inactivation of ARF by AUX/IAA was also upregulated in the leaves. PIN proteins involved in the membrane transport of auxin were also upregulated in roots. Auxin binding protein 1 (ABP1), which is related to the reception of auxin, was explicitly downregulated in the roots.

Jasmonate signalling was the other pathway observed more closely (Figure 6). We found that genes participating in the conjugation of jasmonate acid to isoleucine by JAR1 were uniquely downregulated in leaves. Another unique change in the leaves was the upregulation of JAV1-mediated signalling in the leaves, and 13 DEGs were upregulated during this process (Supplementary Table S10). Only a few DEGs involved in jasmonic acid signalling were found in the roots. These three genes participate in the JAZ-mediated route of jasmonate signalling.

Finally, we further investigated the “response to drought” pathway. Among the up- and downregulated DEGs in the leaf samples, we identified several transcription factors and receptors involved in the “HSFA7/HSFA6B-regulatory network induced by drought and ABA” and ABA-mediated signalling. Interestingly, two aquaporin genes (NP_001234103.1 and NP_001274695.1) were also found among the downregulated DEGs. Among the upregulated genes, similar to the downregulated genes, we identified the subcategory “HSFA7/HSFA6B-regulatory network-induced by drought and ABA”, with several DEGs involved, including those encoding calmodulin (XP_004230637.1) and the putative calcium-binding protein CML19 (XP_004231789.1), heat stress transcription factor A-1b-like isoform X2 (XP_019067864.1), serine/threonine-protein kinase SAPK10 isoform X2 (XP_025885402.1), calcium-binding allergen Ole e 8 (XP_004241666.2), prefoldin subunit 6 (XP_004244801.1), putative transferase At4g12130, mitochondrial isoform X2 (XP_004245713.1), and probable protein phosphatase 2C 10 isoform X1 (XP_010327495.1) (Supplementary Table S10).

In the roots, only a few DEGs were downregulated, including three protein phosphatase 2 C genes (XP_004242517.1; XP_004243737.1; XP_010327495.1) and genes encoding a HIPP transcription factor (XP_019070320.1), a myb-related protein (XP_004249601.1) and an endochitinase (XP_004240090.1) (Supplementary Table S10).

The upregulated genes included genes encoding two reticulon-like proteins (XP_004235819.1; XP_004238895.2), two NAC domain-containing proteins (XP_004244460.1; XP_004246074.1), and two epoxide hydrolases (NP_001316479.1; XP_025886275.1). In addition, a PP2C 50 isoform X5 (XP_010324034.1), the probable calcium-binding protein CML44 (XP_004237355.1), and an uncharacterized protein (XP_025888029.1) were upregulated (Supplementary Table S10).

### 2.6. Expression Analysis of Transcription Factors

**Figure 7:**
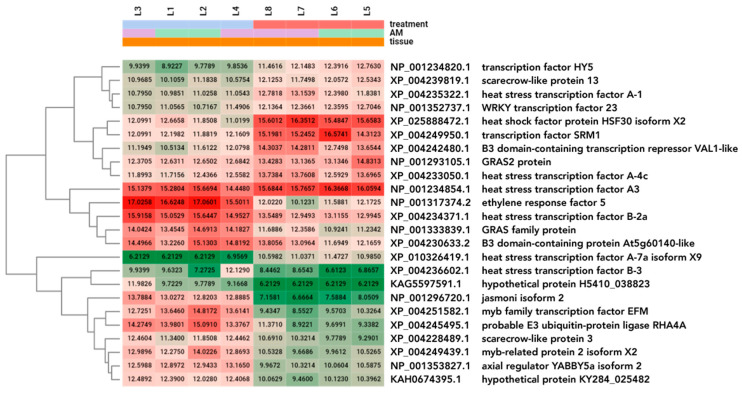
Heatmap of multifactorial DEG analysis of investigated TFs leaf samples. DEGs of TFs indicate TFs that are up- or downregulated by heat stress in the presence of AM. Marks: treatments: red—heat stress; blue—control; purple–AM-inoculated; green—non-mycorrhizal; sample: L1, L2–control leaf samples; L3, L4—mycorrhizal leaf samples; L5, L6—heat stress-treated leaf samples; L7, L8—mycorrhizal and heat stress-treated leaf samples.

Since the global transcriptomic response suggested strong regulatory changes between the tested conditions, we investigated the expression of 107 transcription factors (TFs) that may regulate physiological changes such as hormonal signalling, stress responses, AM fungal accommodation in root cells, arbuscule formation, or nutritional changes (Supplementary Table S9). According to these results, we found only two upregulated sequences in heat-stressed root samples. These genes encoded heat stress transcription factor A-6b (XP_004247676.1) and heat stress transcription factor B-2a-like (XP_004232865.1). In contrast, in the heat-stressed leaf samples, 11 upregulated and 13 downregulated genes were found under the same conditions (Figure 7, Supplementary Table S11). No shared TFs were found in the root and leaf AM samples after exposure to heat stress.

### 2.7. Expression Analysis of Stress Response Enzymes

H_2_O_2_ accumulation was lower in the HAM plants than in the H plants, which suggested that HAM reversed the oxidative stress balance. To obtain more accurate information on the underlying redox state, we directly examined the expression of 304 stress response genes by DEG analysis (Supplementary Table S9).

**Figure 8:**
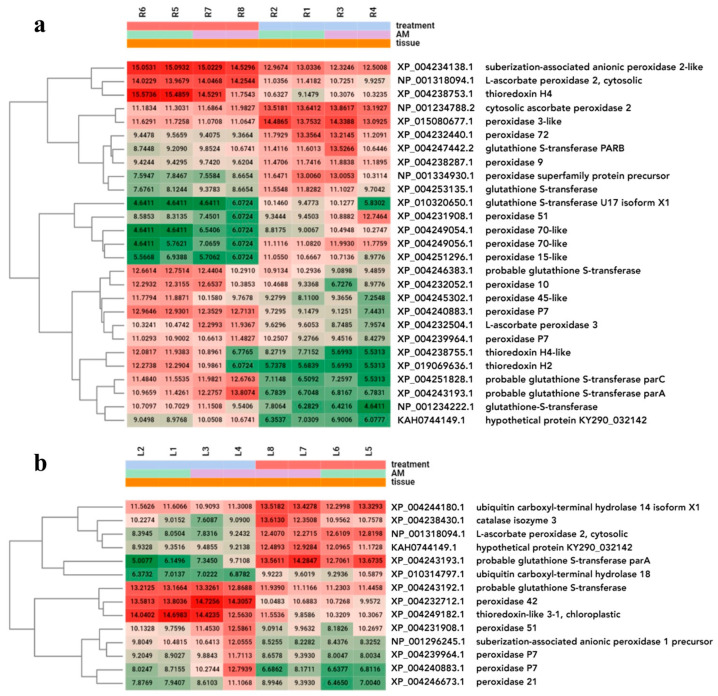
Heatmap of multifactorial DEG analysis of investigated stress genes. Figure shows differentially expressed stress genes that were up or downregulated by heat stress in the presence of AM of root (**a**) and leaf (**b**) samples. Marks: treatments: red—heat stress; blue—control; purple—AM-inoculated; green—non-mycorrhizal; sample: R1, R2—control root samples; R3, R4—mycorrhizal root samples; R5, R6—heat stress-treated root samples; R7, R8—mycorrhizal and heat stress-treated root samples; L1, L2—control leaf samples; L3, L4—mycorrhizal leaf samples; L5, L6—heat stress-treated leaf samples; L7, L8—mycorrhizal and heat stress-treated leaf samples.

Using the same dataset of 304 stress genes, 15 and 12 up- and downregulated genes were detected in the root samples; however, fewer DEGs were detected in the leaf samples (6 upregulated and 8 downregulated). The shared genes were genes encoding L-ascorbate peroxidase 2, cytosolic (NP_001318094.1), peroxidase 51 (XP_004231908.1), peroxidase P7 (XP_004240883.1), peroxidase P7 (XP_004239964.1), probable glutathione S-transferase parA (XP_004243193.1), and ubiquitin carboxyl-terminal hydrolase 18 (XP_010314797.1) (Figure 8, Supplementary Table S11).

### 2.8. Validation of RNA-Seq Results through qRT–PCR

To confirm the RNA-seq expression results, qRT–PCR experiments were performed on five transcripts, namely bidirectional sugar transporter (ST), choline transporter-like protein (CholT), AP2-like ethylene-responsive transcription factor PLT2 (APL2), transcription factor DIVARICATA (MYB), and chaperone protein ClpB (CLPB) (Supplementary Figure S3). The results confirmed the expression trend shown by RNA-seq of the five tested genes, as we found that the genes of interest showed significantly different expressions in the presence of both heat stress and mycorrhizal fungal colonization. The correlation of data from the two experiments can be seen in Supplementary Figure S3. The qRT–PCR and RNA-seq data correlated well with each other.

## 3. Discussion

In our previous work, by comparing the effects of seven mycorrhizal species (*Rhizophagus irregularis*, *Funneliformis mosseae*, *Funneliformis geosporum*, *Funneliformis verruculosum*, *Funneliformis coronatum*, *Septoglomus deserticola*, *Septoglomus constrictum*), we found that *S. constrictum* effectively enhanced tomato tolerance to drought and heat stress [44]. The combined observation of the two stress factors is important because they usually occur simultaneously in nature. Modern agricultural systems enable drought stress mitigation through irrigation, but high temperatures are often unavoidable. With this in mind, we shifted our focus to heat stress alone. In this study, we inoculated tomato plants with *S. constrictum*, as it has been shown in previous studies to be an effective mycorrhizal inoculant that germinates well and is adaptable to different environmental conditions [45,46,47]. Moreover, this strain was among the best-performing strains for enhancing tomato plant tolerance to drought and heat stress in the abovementioned experiment [44].

Heat stress generally causes a considerable reduction in plant biomass [48,49,50]. However, due to the short duration of heat stress and sampling being performed immediately after stress application, physiological changes had not yet occurred at the time of sampling (Supplementary Table S1). On the other hand, the leaf water potential decreased significantly under heat stress, and AM fungal symbiosis did not provide any benefit, as there was no difference between H and HAM plants. This is in accordance with the findings of Duc et al. [12]; however, it is inconsistent with those of other studies, as AM fungi have consistently been shown to improve the water status of plants under different stress conditions, such as heat stress, salt stress, and water deprivation [14,51,52]. In addition to the physiological parameters, the general stress status of tomato plants was observed by measuring the H_2_O_2_ and MDA levels, as these molecules, depending on their cellular level, activate defence responses or initiate cell death after a critical level [53]. ROS, such as superoxide (O_2_^−^) radicals, are actively generated during stress response and are then transformed to H_2_O_2_ in aerobic life forms before finally being neutralized by ROS-scavenging enzymes [54]. One of the main enzymes responsible for H_2_O_2_ production is superoxide dismutase (SOD), which catalyses the dismutation of O_2_^−^ to H_2_O_2_ and O_2_. Interestingly, three SOD genes (NP_001300698.1, NP_001233789.2, and NP_001234769.2) were downregulated in the HAM plants compared to the H plants. The reduced SOD activity might be one of the factors responsible for the decrease in H_2_O_2_ accumulation in heat stress-treated mycorrhizal plants. 

MDA, a toxic reactive aldehyde to cells, is the end product of ROS-mediated degradation of polyunsaturated lipids. The levels of MDA in heat stress-treated nonmycorrhizal and mycorrhizal plants were similar, presumably due to decreased ROS accumulation [55]. This finding is inconsistent with the study of Mathur et al. [13], who reported significantly lower MDA levels in mycorrhizal plants under heat stress than in control plants. However, it must be mentioned that Mathur et al. [13] studied maize, and stress imposition differed markedly between the two experiments. The fact that H_2_O_2_ and MDA behaved differently can be attributed to H_2_O_2_ being an intermediary product during ROS scavenging, while MDA is an end product of the harmful effects.

The lower H_2_O_2_ accumulation in HAM plants compared to H plants suggested that the oxidative stress balance was reversed by mycorrhizal presence. To further look into the molecular background of this difference, 304 stress response genes that mediate the redox state of plants were identified. In both the roots and leaves, peroxidase genes were the most common among the significant DEGs in the HAM plants. Most of these genes were upregulated, consistent with the reduced H_2_O_2_ levels at the molecular level. Increased transcription of peroxidase genes was demonstrated in previous AM-related studies [37,42]. The GST genes were also strongly influenced by the HAM treatment. Among these genes, glutathione S-transferase U17 isoform X1 (XP_010320650.1), which was detected in leaves, was the most interesting because it seemed to be consistently detected in every AM-related tomato study utilizing RNAseq [37,38,56]. 

Among the top 50 differentially expressed genes (ranked by FDR) across all our leaf and root samples, heat shock factor and proteins were found in the greatest numbers (Figure 3). These genes were differentially expressed not only in the leaves but also in the roots. Heat stress activates the heat stress signal transduction network of HSFs, which modulates the heat stress response. In addition, 27 heat stress transcription factors that participate in thermotolerance regulation have been reported in tomato [57,58,59,60]. A wide range of genes involved in the signalling and metabolic pathways of the heat shock response, such as heat shock proteins (HSPs) and ROS-scavenging enzymes, were induced [61]. HSP100, HSP90, HSP70, HSP60, and small HSPs function as molecular chaperones that stabilize and renature misfolded proteins [27].

Altogether, the mycorrhizal priming of ROS scavenging enzyme transcription results in more resilience against harmful environmental factors. Moreover, the higher induction of the chaperon and HSF genes results in the translation of the necessary proteins for thermotolerance as misfolded proteins are corrected or ubiquitinated and directed to the proteasome for degradation. Through these changes, plant growth is hindered at a lower level in mycorrhizal plants.

Fiorilli et al. [62] used a TOM2 microarray to acquire an overview of the transcriptional changes induced by *Funneliformis mosseae* in tomato plants. These authors found that the most responsive genes were involved in primary and secondary metabolism, defence and response to stimuli, cell organization and protein modification, and transcriptional regulation. Either these GO categories or their sub- or supercategories were also found in our experiment (Supplementary Figure S4). Interestingly, photosynthesis-related GO terms were enriched in all the HAM comparisons in leaves. The genes included in these categories, such as those encoding chlorophyll a-b binding protein, were, without exception, downregulated in the heat-stressed mycorrhizal plants compared with the other treatments. Mycorrhizal fungi stimulate plant photosynthesis to satisfy plant and fungal carbon needs [63,64]. In the presence of heat stress, to reduce evaporation [65], photosynthetic activity decreased, and this downregulation was surprising because AM has been shown to promote photosynthesis even under stress conditions [31].

GSEA showed that transport localization and protein modification were induced across all comparison pairs, indicating that the co-occurrence of mycorrhizal fungus and heat stress significantly induced this effect. Amino acid, oxoacid, organic acid, carboxylic acid metabolic processes, and nucleolus and ribosome–related GOs were lacking only in HAMvsH in leaves, suggesting that the synthesis of macromolecules containing organic acids may be stronger in the roots, and the products may be transported to the leaves when AM symbionts are present. By focusing more on the GSEA data of HAMvsH by filtering more strictly, we found that ribosome- and translation-related processes were enhanced in the mycorrhizal leaves compared to nonmycorrhizal leaves during heat stress. In the work of Yangueez et al. [66], these processes were preferentially downregulated to save energy and prevent the accumulation of misfolded proteins due to heat stress in Arabidopsis [66]. This indicates that the presence of AM could reduce the heat stress-induced decrease in protein synthesis in leaves.

A total of 107 transcription factors related to mycorrhizal colonization or heat stress identified in the literature were compared to our results. Unsurprisingly, 7 of the 27 tomato HSF genes were found to be differentially expressed in leaves under heat stress. In addition to HSF, TFs belonging to the GRAS family, members of the AP2/ERF, bZIP and MYB families were identified among the DEGs; these genes influence developmental changes, growth, and responses to environmental stresses. The expression of ethylene response factor 5 (NP_001317374.2) was downregulated in our study. Both the up- and downregulation of this TF by different mycorrhizal strains and the downregulation of this TF by heat stress have been demonstrated in tomato [7,38,42,56]. Our results further confirmed the mycorrhizal inducibility of this gene.

To identify mycorrhizal fungus-specific changes during heat stress, we examined and compared the DEGs enriched in HAMvsH with CAMvsC and further observed only those that were found in the former but not in the latter (Figure 6). Interestingly, most of the DEGs were unique to HAMvsH or CAMvsC in both sample types regardless of the up- or downregulation of the genes, and only a few genes overlapped between the two comparisons. The ratio of DEGs in the leaves to that in the roots remained similar.

Pathway analysis of unique mycorrhiza-induced DEGs in HAMvsH was also performed. Interestingly, drought stress response genes were among the DEGs identified in both the root and leaf samples. Drought and heat stress can have some overlapping effects on plants, as both conditions can lead to dehydration and adversely affect various physiological processes. This overlap has already been demonstrated previously [67]. The Plant Reactome Database does not contain a well-defined heat stress signalling pathway, which has led to the identification of a related pathway, the drought response pathway. Several genes in the subcategory “HSFA7/HSFA6B-regulatory network induced by drought and ABA” were upregulated in both the roots and leaves, with more DEGs observed in the leaf samples. HSFA6b and ABA signalling have been connected to thermotolerance [68]. This network seems to be strongly influenced by systemic mycorrhizal colonization, as several of the participants in this pathway were differentially expressed.

Auxin signalling genes were abundant in our HAM samples, so this pathway was also selected for further investigation. It regulates diverse physiological processes, including cell elongation, root initiation, apical dominance, tropic responses, and vascular tissue development [69]. This pathway also plays a crucial role in the interplay between AM fungi and their host, both in the early stage of colonization by promoting the establishment of colonization and in later stages of colonization by modulating AM fungus-promoted root growth [37,70]. Auxin also plays an important role in heat stress-induced thermomorphogenesis [71]. According to our results, the transition of plant growth during heat stress is strongly influenced by the AM fungus through the modification of auxin transport and signalling throughout the whole plant.

We examined the DEGs associated with jasmonate signalling, another hormone signalling pathway (in addition to auxin signalling). Jasmonates regulate heat stress-responsive genes, modulate antioxidant systems, and stimulate the production of secondary metabolites [72]. Jasmonate-mediated plant defence-related mechanisms play a major role in establishing and controlling symbioses [73]. Upon the manifestation of symbiotic interactions, AM fungus modulates jasmonic acid production and helps plants respond to abiotic and biotic stresses [74]. The participation of DEGs in jasmonic acid signalling, especially in JAV1-mediated signalling in leaves, was upregulated in HAM plants. As JAV1 is one of the main controllers of the development of plant stress tolerance [74], it is suggested to play a role in the AM fungus-induced beneficial effects.

All three of the abovementioned pathways indicate a strong phytohormone metabolism difference in the response of AM and non-AM plants to heat shock. 

As our findings are only exploratory, further investigations should be performed to provide a more comprehensive understanding of these biological systems. One option could be to utilize the other branches of omics, proteomics and metabolomics, to confirm if the molecular changes observed by us do manifest at higher levels as well. The changes in the hormonal pathways highlighted in our study can be further investigated with the help of hormonal pathway mutant plants. Moreover, functional assays of the genes found to be induced in mycorrhizal heat stress-treated plants in order to confirm their specified role in AM-induced heat resistance. 

## 4. Materials and Methods

### 4.1. Plant Material

*Septoglomus constrictum* (provided by Prof. Janusz Blaszkowski, Department of Plant Protection, West Pomeranian University of Technology, Szczecin, Poland) isolates were propagated for 6 months in sterilized sand using *Zea mays* L. var. saccharata and *Medicago truncatula* L. as host plants. The inoculant acquired this way contained mycelia, spores, colonized root fragments from *S. constrictum*, and substrate (sterilized sand). Tomato (*Solanum lycopersicum* L.) var. Moneymaker seeds commercially bought were surface sterilized with 2.5% sodium hypochlorite containing 0.02% (*v*/*v*) Tween-20 for 30 min, rinsed with distilled water, and germinated in a Petri dish in the dark at 24 °C for 3 days. Germinated seeds were transferred into half-litre plastic pots (one seed/pot) filled with 0.5 kg of a sterilized mixture of sand and peat (4:1, *v*/*v*, Klasmann TS3, P_2_O_5_ 0.1 g L^−1^). Mycorrhizal inoculation was performed at the time of the planting for each pot with 50 g (30 spores g^−1^) of the sand containing the previously described inoculum, while plants without AM fungal treatment were supplemented with 50 g of three-times-sterilized (121 °C for 60 min) inoculum. After 2 weeks of growth, the homogeneity of the seedlings was checked and the final participants of the experiment were chosen. The final experiment included 24 pots of non-AM plants and 24 pots of plants inoculated with *S. constrictum*. Plants were arranged randomly and grown in a climate chamber at 26/20 °C (16/8 h), a photosynthetic photon flux density (PPFD) of 600 µmol m^−2^ s^−1^, and relative humidity (RH) of 60% for 4 more weeks. Stress was then applied on the 42nd day. Half of the AM and non-AM plants were moved to a different climate chamber and treated with a 6 h long 45 °C heat shock immediately before harvest. Altogether, four treatments were set up from the original 24 AM and 24 non-AM plants in twelve replicates: mycorrhizal (CAM) and non-mycorrhizal non-stress control (C) plants, and heat-stressed mycorrhizal (HAM) and non-mycorrhizal (H) plants. Plant material (complete leaves of the second leaf level from the top and complete root) for stress marker and molecular measurements were immediately frozen after heat shock treatment in liquid nitrogen and stored at −80 °C.

### 4.2. Mycorrhizal Colonization Assessment

The roots of three biological replicates were washed with tap water, cleared with 10% KOH for 10 min, and acidified with 2% hydrochloric acid. Staining was performed overnight with 0.05% trypan blue (1:1:1 proportion of water/glycerol/lactic acid) [75]. The mycorrhizal colonization rate was determined using the gridline intersection method by observing fifty root pieces under a stereo microscope [76].

### 4.3. Shoot and Root Dry Weight, and Leaf Water Potential Measurement

The fresh shoot and root weight of three biological replicates were weighed at the time of harvesting after those plants had been dried in a hot-air oven at 70 °C until a constant weight was reached. Leaf water potential was measured by a pressure chamber, following the methodology of Boyer [77] and using three plants per treatment.

### 4.4. Hydrogen Peroxide and Malondialdehyde Content Measurements

Hydrogen peroxide (H_2_O_2_) content was quantified according to the method of Alexieva et al. [78] for all treatments from leaf samples of four biological replicates. Fresh leaf samples (500 mg) were grounded in 5 mL of cold 0.1% (*w*/*v*) trichloroacetic acid (TCA) and then centrifuged at 12,000× *g* for 15 min at 4 °C. Leaf extract supernatant (0.5 mL) was added to 0.5 mL of 100 mM potassium phosphate buffer (pH 7.0) and 1 mL of 1M KI, and left in the dark for 1 h. Absorbance was measured at 390 nm (UV–VIS spectrophotometer U-2900, Hitachi, Japan).

Lipid peroxidation level was measured by the method of Heath and Packer [79] for all treatments from leaf samples of four biological replicates. Fresh leaf samples (500 mg) were ground in 5 mL 0.1% TCA and then centrifuged at 10,000× *g* for 5 min. A 1 mL aliquot of the leaf supernatant was added to 4 mL 20% TCA containing 0.5% 2-thiobarbituric acid and was incubated at 95 °C for 15 min. The mixture was cooled immediately after incubation time. Malondialdehyde (MDA) content was estimated by subtracting the non-specific absorption at 600 nm from the absorbances at 532 nm; the absorption coefficient was 155 mM^−1^ cm^−1^.

### 4.5. RNA Isolation

For RNA sequencing, total RNA was extracted from root and leaf samples of two biological replicates from each treatment using an EZNA Plant RNA Kit (Omega Bio-Tek, Norcross, GA, USA). Samples (100 mg) were processed during isolation following the manufacturer’s protocol. After isolation, RNA integrity was examined with an Agilent 2100 Bioanalyzer.

### 4.6. Preprocessing of RNA-Seq Reads

Libraries were sequenced with a final output of single-end reads, as detailed in Supplementary Table S3. Quality control (QC), trimming, and filtering of fastq files were performed in a preprocessing step. The QC analysis was performed with FastQC software v0.12.1 [80]. The Phred-like quality scores (Q scores) were set to >30. Poor quality reads, adapters at the ends of reads, and limited skewing at the ends of reads were eliminated by using Trimmomatic [81]. Contamination sequences and Ns were filtered out with a self-developed application GenoUtils as described earlier [82]. Reads containing Ns more than 30% were eliminated, while reads with a lower N’ ratio were trimmed with a final length > 65. Reads that passed preprocessing were further mapped to the reference genome (GCF_000188115.5) and analysed.

### 4.7. Mapping of RNA-Seq Reads to the Reference

Cleaned reads were mapped to the reference genome dataset by using Bowtie 2 (downloaded from NCBI 05.13.2023, GCF_000188115.5_SL3.1). Those CDS sequences and reads that were aligned were kept and analysed further. Re-annotation of kept CDS sequences was performed using Blast (Blastx, viridiplantae database), GO Mapping, GO Annotation, and functional analysis (GO-Slim). The Interpro database was used to link GO terms to genes. Diamond Blast alignment was performed against the NR (10 May 2023) database, using a viridiplantae (33090) taxonomic filter.

### 4.8. Gene-Level Quantification and Differential Expression Analysis of Genes (DEGs)

We aligned sequencing reads to estimate gene expression from RNA-sequencing experiments and kept the results in coordinates with genomic features. The abundance matrix of reads mapped to CDS sequences was created for further analysis. Only reads mapping unambiguously to a single genomic feature were considered. Reads aligned to more than one position or overlapping with more than one feature were discarded. This step was performed using the HTSeq package [83]. 

To identify genes expressed in significantly different quantities in distinct groups of samples, pairwise differential expression analysis was performed using the program NOISeq [84,85] which can compare samples from two experimental conditions by simulating replicates if the biological and technical replication is small (in the present experiment only 2 replicates were available per sample). The gene expression index Reads Per Kilobase per Million Mapped reads (RPKM) was used in normalization processes.

Genes that were differentially expressed were identified in the samples of leaf and root separately by performing pairwise comparisons of mycorrhizal heat stress-treated to control (HAMvsC), mycorrhizal heat stress-treated to mycorrhizal control (HAMvsCAM), and mycorrhizal heat stress-treated to heat stress-treated (HAMvsH) plants. The differential expression analysis was combined with enrichment analysis of sample pairs. 

To the global visualization of the level of similarity of individual datasets, the MDS plot (multidimensional scaling plot) was selected to “translate information” about the pairwise ‘distances’ between the paired samples involving root and leaf combined and separated analysis (Supplementary Figure S2). 

The numerical overview of up- and downregulated genes in samples is shown in the Venn diagrams. Unique genes of pairwise samples were analysed functionally to determine the GO terms. According to the information deriving from transcriptomic alterations caused by mycorrhiza symbiosis, we analysed the sample pair HAM vs. H in both leaf and root samples.

The top 50 DEGs of combined (leaf and root) DEG datasets were visualized in a two-dimensional heatmap (Figure 3). The results of all pairwise DEGs are presented in Supplementary Tables S4 and S5.

### 4.9. Expression Analysis of Transcription Factors and Stress Genes

The investigated 107 transcription factors (TFs) are detailed in Supplementary Table S9. DEG analysis was performed for these selected TFs. The configuration of pairwise DEG analysis was a multifactorial design, where primary and secondary factors were AM and heat stress. The GLM (Quasi Likelihood F-Test) statistical test was applied using edgeR, [86]. 

The list of 304 stress genes tested is shown in Supplementary Table S9. DEG and statistical analysis for stress genes were performed as described above. The underlying RPKM value of the entire dataset is presented.

### 4.10. Enrichment Analysis

Functional enrichment analysis from the pairwise data was performed by Fisher’s exact test [87] and gene set enrichment analysis (GSEA) [88].

Fisher’s exact test was used to find GO terms that are over and under-represented in a set of genes (test set) concerning the reference group (reference set). This test was used to analyse the filtered genes based on Venn diagrams. GSEA was used to analyse the ranked gene list of DEGs using the following formula:

Rank = sign(logFC) × (−log10(*p*-Value))

where logFC is the measure that describes how much the expression changes between conditions (log2-fold-changes). GSEA analysis was performed with root and leaf HAM vs. (H, C, and CAM) sample pairs. Following Subramanian et al. [88], we determined core and non-core enriched genes. The core enriched sequences are the ones that contribute the most to the enrichment of a particular function. GSEA analyses were evaluated according to the normalized enrichment score (NES).

### 4.11. Pathway Analysis

To get an overview of the biological mechanisms involved in the data of pairwise DEGs and summarize the background information of molecular mechanisms, pathway analysis of DEGs was performed using the KEGG and Plant Reactome databases [89,90] by using omicsbox biobam (Version 1.4.337). We applied the pathway analysis to compare the characteristic genes found when comparing HAMvsH and CAMvsC sample pairs.

### 4.12. Quantitative RT-PCR

The RNA samples sent for RNA sequencing were supplemented with total RNA isolates from two more biological replicates, resulting in a total of four replicates used in the RT-PCR measurements. DNaseI (Thermo Scientific, Waltham, MA, USA) was applied to the samples. RNA integrity was evaluated according to the 260/280 and 260/230 nm absorbance ratios measured by a Nanophotometer (IMPLEN GmbH, München, Germany). cDNA was synthesized from 0.5 µg of RNA by a RevertAid First Strand cDNA Synthesis Kit (Thermo Scientific, Waltham, MA, USA). Before quantitative real-time PCR experiments, the cDNA was diluted 1:5. Quantitative real-time PCR amplification reactions were performed on 96-well plates on a Stratagene Mx3000P Real-Time PCR System using ABsolute SYBR Green Low ROX qPCR Mix (Thermo Fisher Scientific, Inc., Vilnius, Lithuania), with three technical replications. The PCR mixtures consisted of 12.5 μL of ABsolute SYBR Green Low ROX qPCR Mix, 1 μL of each forward and reverse primer (10 mM), and 9.5 μL of nuclease-free water. Finally, 1 μL of diluted template cDNA (1:5) was added, resulting in a total volume of 25 μL PCR mixture-1. The PCR cycling program was as follows: 15 min at 95 °C followed by 40 cycles of 15 s at 95 °C, 30 s at 59 °C, and 20 s at 72 °C. Melting curve cycling consisted of 60 s at 95 °C, 30 s at 59 °C, and 30 s at 95 °C. The relative transcript abundance was determined by normalization to the abundance of the internal control elongation factor 1-alpha (Ef1α) and actin genes as these are commonly used reference genes in tomato research [91] and have been shown to be stable under mycorrhizal inoculation, and in the case of some abiotic factors [92,93], with the use of the 2−ΔΔCt method [94]. For primer sequences, see Supplementary Table S2.

### 4.13. RNAseq-qRT-PCR Comparison

Four biological replicates were used for the qRT-PCR and two for the RNAseq, the average of which has been used in a Pearson correlation analysis. The expression of the following genes was compared: ST (bidirectional sugar transporter, XM_004235291.4), Cholt (choline transporter-like protein, XM_004244174.4), AP2L (AP2-like ethylene-responsive transcription factor PLT2, XM_004231900.4), MYB (transcription factor DIVARICATA, XM_004235209.4), and CLPB (chaperone protein ClpB, XM_010323592.3) (Supplementary Figure S3).

### 4.14. Statistical Analysis

Plant biomass data, leaf water potential, H_2_O_2_ and MDA content, and gene expression measurements were statistically analysed using R statistical software 4.0.2 [95]. Normality and homoscedasticity were checked via Kolmogorov–Smirnov and Levene tests, respectively. One-way analysis of variance (ANOVA) was used, and the means were compared using Tukey’s post hoc test. A *p*-value < 0.05 was considered to indicate statistical significance.

## 5. Conclusions

Tomato plants inoculated with *Septoglomus constrictum* seemed to be more resilient to heat-induced oxidative stress. The lower levels of H_2_O_2_ parallels well to the downregulation of SOD genes in HAM treatments and the higher expression of several peroxidase and glutathione glutathione S-transferase genes. Among the pathways and genes in which mycorrhizal plants differed the most from their non-mycorrhizal counterparts during heat stress, we found jasmonate and auxin signalling, and genes overlapping with drought stress response, especially abscisic acid-responsive genes, in both the root and leaf samples. As we hypothesized, these indicate *S. constrictum* has a strong influence on the oxidative stress elimination and phytohormone metabolism of tomato at a transcriptomic level. Besides the direct stress response, sugar, lipid, and protein metabolism and transport processes are all influenced by the mycorrhizal fungus, resulting in the alleviation of stress imposed by high temperatures. Therefore, our study provides useful datasets and candidate genes related to the stress response of mycorrhizal tomato plants under heat stress for further studies. However, we propose that the observed changes be examined in a more focused manner by using jasmonate or auxin tomato mutants, or by utilizing proteomics and metabolomics as well, because our study contains only preliminary exploratory results on the transcriptomic level.

## Data Availability

The datasets generated and/or analysed during the current study are available in the NCBI database/BioProject/PRJNA1085239. https://www.ncbi.nlm.nih.gov/bioproject/PRJNA1085239/ (created on 7 March 2024).

## Supplementary Materials

The following supporting information can be downloaded at: https://www.mdpi.com/article/10.3390/plants13162266/s1, Table S1. Plant dry biomass, relative water content, and colonization results; Table S2. Primer sequences used for qRT-PCR; Table S3. Numerical data of Illumina reads of investigated samples after read preprocessing; Table S4. DEGs of treatment comparisons in leaf samples; Table S5. DEGs of treatment comparisons in root samples; Table S6. Data of Figure 5. Venn diagram; Table S7. Top and bottom GSEA results of leaf and root HAMvsH sample pair; Table S8. Core and non-core genes of selected GSEA categories in root HAMvsH samples; Table S9. DEG analysis of the 107 transcription factors (TFs) and 304 stress genes; Table S10. Response to drought, Jasmonic acid signalling, and Auxin signalling process pathways; Table S11. The available literature from the investigated transcription factor and stress enzyme genes showed a significant expression change, detailed in Figure 7 and Figure 8 [96]. Figure S1. Two-dimensional scatterplot in which the distances represent the log2 fold changes between samples; Figure S2. Plant-reactome pathway analysis of uniquely expressed genes in R7, R8, and L7, L8 samples; Figure S3. Correlation analysis of qRT-PCR and RPKM values; Figure S4. GO term determination based on Fisher’s exact test of unique DEGs of HAM vs. H sample pairs in root and leaf samples.
