# Supplementary material for: First Peek into the Transcriptomic Response in Heat-Stressed Tomato Inoculated with Septoglomus constrictum
Source: Plants (Basel). 2024 Aug 15;13(16):2266. doi: 10.3390/plants13162266 (PMC11359853; doi:10.3390/plants13162266)
Supplement: Supplementary file 1 [file plants-13-02266-s001.zip › Szentpeteri_et_al. Supplementary Figures.pdf]

First Peek into the Transcriptomic Response in Heat Stressed Tomato inoculated with *Septoglomus constrictum*

Supplementary Figures

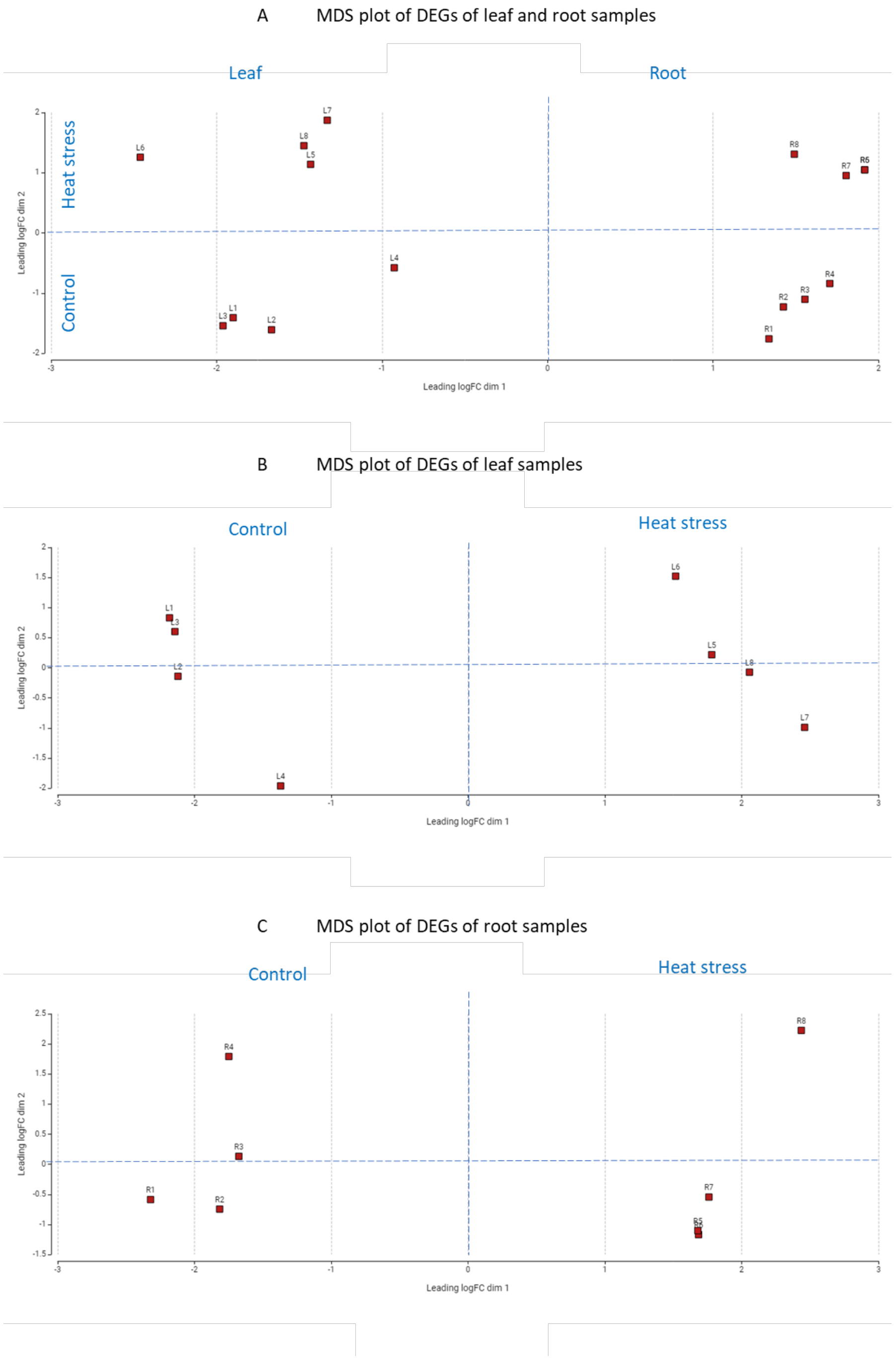

Supplementary Figure S1. Two-dimensional scatterplot in which the distances represent the log2 fold changes between samples. Comparison of all root and leaf samples (A) and separated analysis of the leaf (B) and root (C) samples.

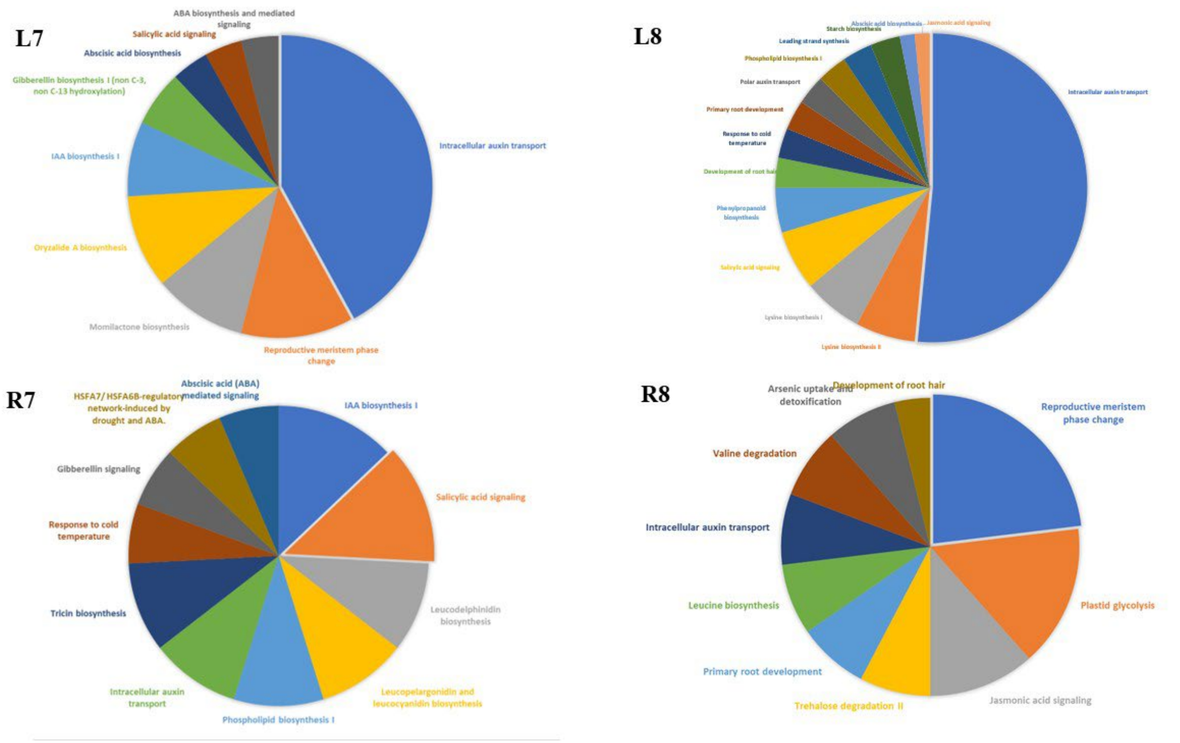

Supplementary Figure S2. Plant-reactome pathway analysis of uniquely expressed genes in R7, R8 and L7, L8 samples. The first 10 pathways are visualized filtered to *Solanum lycopersicum* pathway ID (R-SLY).

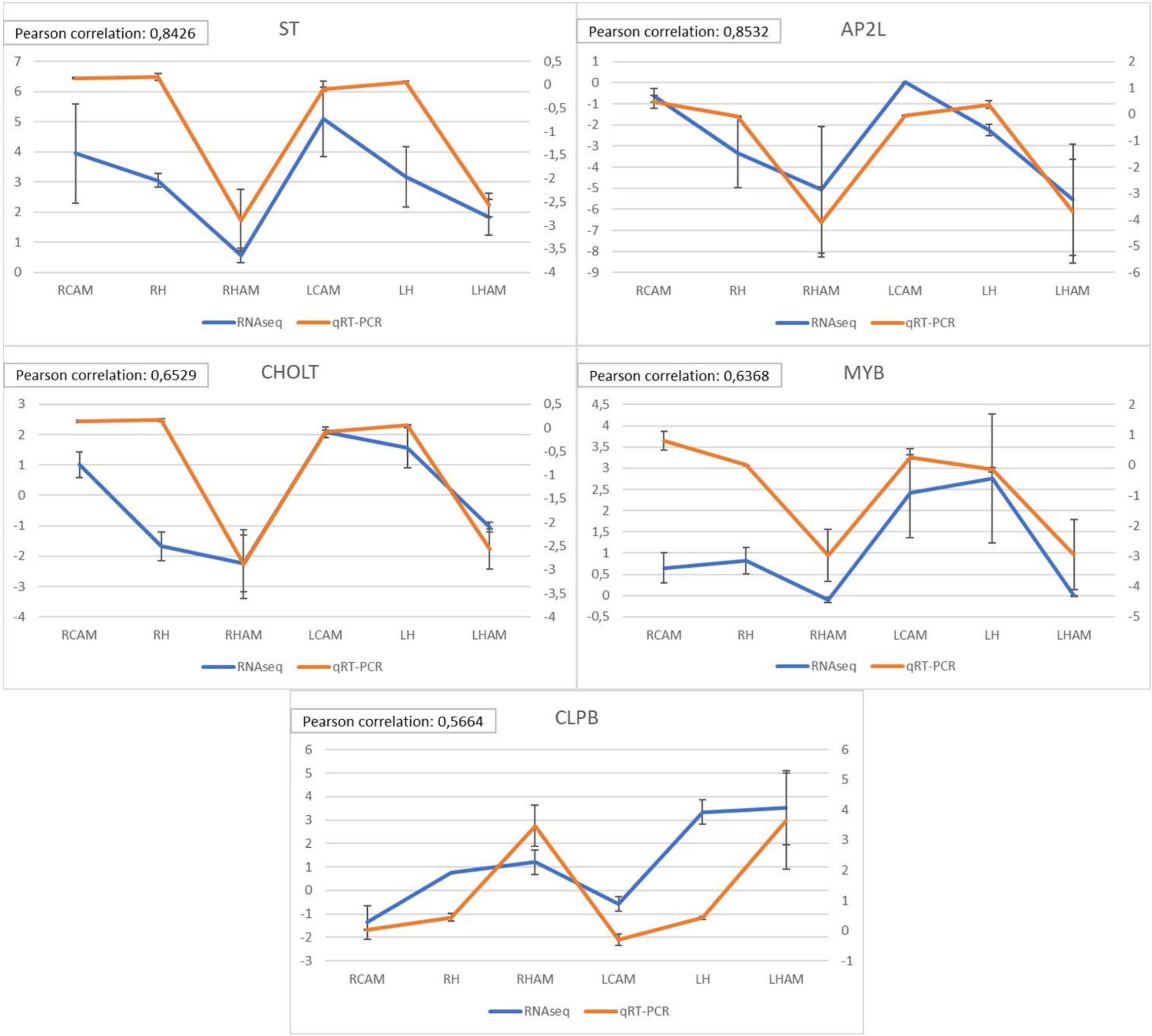

Supplementary Figure S3. Correlation analysis of qRT-PCR and RPKM values. The average of four biological replicates were used for the qRT-PCR and two for the RNAseq

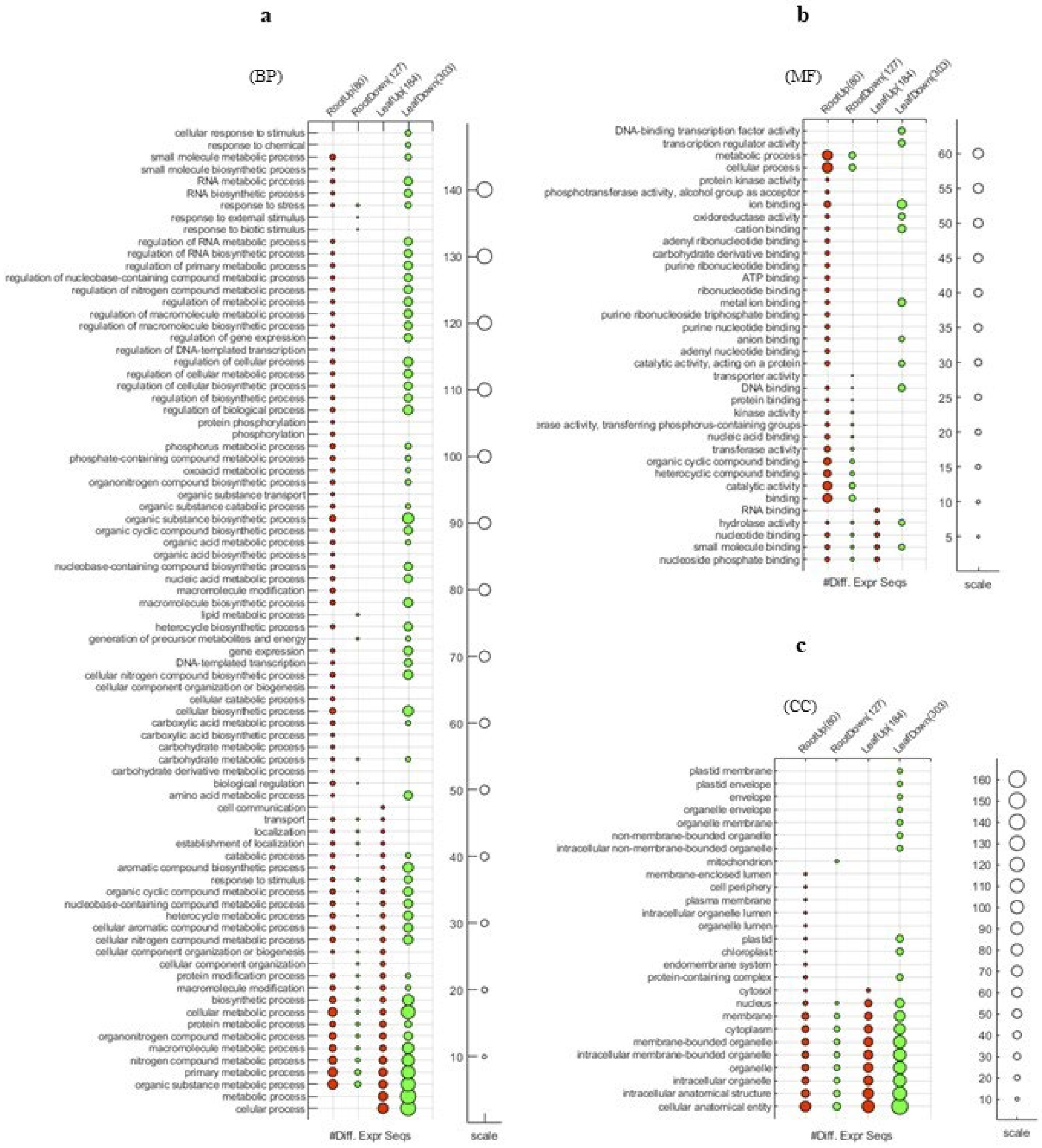

Supplementary Figure S4. GO term determination based on Fisher's exact test of unique DEGs of HAM vs H sample pairs in root and leaf samples. The number of found and GO-categorized sequences showing differential expression are illustrated by dot sizes and compared in the investigated tissues. A: biological process (BP); B: molecular function (MF); C: cellular component (CC).
